# Prosthetic Joint Infection Trends at a Dedicated Orthopaedics Specialty Hospital

**DOI:** 10.1155/2019/4629503

**Published:** 2019-02-10

**Authors:** Robert P. Runner, Amanda Mener, James R. Roberson, Thomas L. Bradbury, George N. Guild, Scott D. Boden, Greg A. Erens

**Affiliations:** ^1^Emory University Department of Orthopaedics, Atlanta, GA, USA; ^2^Emory University School of Medicine, Atlanta, GA, USA

## Abstract

**Introduction:**

Historically, a majority of prosthetic joint infections (PJIs) grew Gram-positive bacteria. While previous studies stratified PJI risk with specific organisms by patient comorbidities, we compared infection rates and microbiologic characteristics of PJIs by hospital setting: a dedicated orthopaedic hospital versus a general hospital serving multiple surgical specialties.

**Methods:**

A retrospective review of prospectively collected data on 11,842 consecutive primary hip and knee arthroplasty patients was performed. Arthroplasty cases performed between April 2006 and August 2008 at the general university hospital serving multiple surgical specialties were compared to cases at a single orthopaedic specialty hospital from September 2008 to August 2016.

**Results:**

The general university hospital PJI incidence rate was 1.43%, with 5.3% of infections from Gram-negative species. In comparison, at the dedicated orthopaedic hospital, the overall PJI incidence rate was substantially reduced to 0.75% over the 8-year timeframe. Comparing the final two years of practice at the general university facility to the most recent two years at the dedicated orthopaedics hospital, the PJI incidence was significantly reduced (1.43% vs 0.61%). Though the overall number of infections was reduced, there was a significantly higher proportion of Gram-negative infections over the 8-year timeframe at 25.3%.

**Conclusion:**

In transitioning from a multispecialty university hospital to a dedicated orthopaedic hospital, the PJI incidence has been significantly reduced despite a greater Gram-negative proportion (25.3% versus 5.3%). These results suggest a change in the microbiologic profile of PJI when transitioning to a dedicated orthopaedic facility and that greater Gram-negative antibiotic coverage could be considered.

## 1. Introduction

In the last decade, the demand for hip and knee arthroplasty has grown more than 150% and is expected to continue to grow, with a projected 673% increase in demand for primary total knee arthroplasty by 2030 [1]. With this increase in hip and knee arthroplasty, there will likely be a corresponding increase in postoperative infections. The current infection rate for joint arthroplasty ranges from 0.6% to 2.4% [2]. Furthermore, within two years of knee arthroplasty, the rate of infection is estimated at 1.55% [3]. These postoperative infections can have debilitating consequences for patients, often necessitating extensive antibiotic use with potential side effects and repeat operations. The purpose of this study was to identify and compare the incidence of prosthetic joint infection (PJI) and characterize the microbiologic profile of these infections that occurred between two hospital settings: a general university hospital and a dedicated orthopaedic specialty hospital.

Gram-positive bacteria have been most commonly implicated in PJIs [4–6]. To combat surgical site infections, routine use of prophylactic antibiotics has been implemented; however previous studies have shown that there is a lack of consensus regarding the optimal protocol for prophylactic antibiotic use. According to the Surgical Care Improvement Project Advisory Committee and the American Academy of Orthopaedic Surgeons, the preferred antibiotic prophylaxis for patients undergoing hip or knee replacements is cefazolin or cefuroxime [7, 8]. Cephalosporins are commonly used as antibiotic prophylaxis given their bactericidal activity against Gram-positive and Gram-negative organisms [9]. Previously, it has been shown that the timing of prophylactic antibiotics was critical with the lowest infection rate occurring if antibiotics were administered within one hour before surgery [10, 11]. Furthermore, in addition to the timing of antibiotics, other indications for antibiotic use must be considered, such as the prevalence of resistant strains in the surrounding population. Because of the increased incidence of methicillin-resistant* Staphylococcus aureus* [12, 13], the use of vancomycin has increased. However, the routine use of vancomycin to potentially prevent surgical site infections is a controversial topic. It has previously been shown that diabetes, chronic steroid use, and obesity are risk factors of PJI [14–24] but the root cause of PJI is multifactorial. Additionally, infection season and month has been correlated to an increased risk of PJIs, with summer months accounting for more PJIs compared to winter months [25, 26]. Overall, there is not a clear understanding of the specific factors that contribute to postoperative infections, nor a defined method for preventing these infections from occurring.

Historically, at large academic settings, orthopaedic procedures have been performed alongside other surgical specialties in the same operating rooms. However, recently, there has been a shift towards more dedicated orthopaedics units within general hospitals and even dedicated orthopaedics hospitals. Dedicated orthopaedic units have been associated with decreased complication rates [27, 28]. Even the establishment of a dedicated orthopaedic trauma room at general university hospitals has resulted in a reduction in after-hours surgery and complications [29, 30], improving efficiency and morbidity and mortality. However, less is established in the literature regarding the impact of transitioning to a dedicated orthopaedic hospital in potentially reducing PJIs, improving efficiency, and minimizing complications. The purpose of this study was to compare the infection rates and microbiologic characteristics between a general university and a dedicated orthopaedic specialty hospital in primary arthroplasty patients. The null hypothesis was that the infection rate and microbiologic profile between the institutions would be similar.

## 2. Materials and Methods

Institutional Review Board approval was obtained for this retrospective review of prospectively collected data on 11,842 consecutive primary hip at knee arthroplasty patients performed at a single academic residency program. Arthroplasty cases performed between April 2006 and August 2008 at the general university hospital serving multiple surgical specialties were compared to cases at a single dedicated orthopaedics specialty hospital from September 2008 through August 2016. As the surveillance program at the general university hospital only started in 2006, arthroplasty cases before this date were excluded.

As the original start of data collection was prior to current definitions of PJI [31–33], the definition of infection was kept consistent throughout data collection for both hospitals as all primary arthroplasty patients required return to the operative room for debridement of deep infection within one year of index procedure.

Operative variables examined were as follows: procedure date, whether a resident or fellow was scrubbed on the case, and surgery time. Surgery time was defined in minutes from incision start to surgery end time. Demographic variables collected were as follows: age, gender, smoking history, diabetes status, and history of steroid use. Infection variables on the cultured organism identified (if any), bacterial species, and Gram-negative or Gram-positive status were obtained.

### 2.1. Statistical Analysis

To evaluate differences between the general university hospital and dedicated orthopaedics specialty hospital categorical variables were analyzed with X^2^ analysis. For continuous variables of age and BMI, Mann–Whitney* U* nonparametric analysis was utilized.* p*<0.05 was the cutoff for significance. All statistical analysis was performed in GraphPad Prism (San Diego, CA),

## 3. Results

To begin comparing the infection rates and microbiologic characteristics between the general university hospital and dedicated orthopaedics specialty hospital, the incidence rate of infections in each hospital setting was calculated. The general university hospital PJI incidence rate was 1.43%. In comparison, at the dedicated orthopaedics specialty hospital, the overall PJI incidence rate was substantially reduced to 0.75% (*p*=.0224) (Table 1). When comparing the final two years of practice at the general university facility to the most recent two years at the dedicated orthopaedics specialty hospital, the PJI incidence was significantly reduced from the 1.43% incidence at the general university hospital to 0.61% infection rate at the dedicated orthopaedics specialty hospital (*p*=.0087) (Table 2), with a decrease in the length of surgery for infected cases occurring at the dedicated orthopaedics specialty hospital compared to the general university hospital (Table 3).

To identify differences in infection rates between the general university hospital and dedicated orthopaedics specialty hospital, demographic variables for all PJIs (hip and knee combined) were analyzed, as obesity, history of steroid use, and diabetes have all been previously identified as risk factors to PJIs [15–24].  Table 4 identifies demographic variables between patients with PJIs at both the academic general and dedicated orthopaedics specialty hospital. There was no significant difference in the average age (64.4 versus 61.6 years,* p* =0.13) or BMI (30.5 versus 31.5,* p* =0.52) between the groups. Additionally, there were no statistically significant differences in the proportion of males and females with all PJIs at the academic general and dedicated orthopaedics specialty hospital (*p*=.043), nor were there differences in the proportion of diabetic patients (*p* =.40), proportion of procedures with a resident/fellow scrubbed (*p*=0.67), positive history of steroid use (*p*=0.31), nor smoking history (*p* =0.37) between these two populations. In subgroup analysis of TKA patients based on hospital location, there was no difference in the proportion of males and females (*p*=0.90), diabetic patients (*p*=0.13), nor history of steroid use (*p*=0.09) in knee PJIs at the general university hospital nor dedicated orthopaedics specialty hospital (Table 5). Although there was an observed difference in the proportion of cases with a resident/fellow scrubbed (*p*=0.02) and smoking status (*p*=0.04), we interpret these findings to be reflective of the small number of knee PJIs at the general university hospital (n=8) compared to the dedicated orthopaedics specialty hospital (n=35), as well as the significant proportion of nonreported smoking statuses at the general university hospital (12.5%) (Table 5). Additionally, there was no difference in the proportion of males and females (*p*=0.30), diabetic patients (*p*=0.83), whether a resident or fellow was scrubbed (*p*=0.15), history of steroid use (*p*=0.56) nor smoking status (*p*=0.24) in THA PJI (Table 6). These results indicate that, despite the decreased incidence of infections at the dedicated orthopaedics specialty hospitals, the overall baseline characteristics of the patient populations were mostly similar.

**Figure 1:**
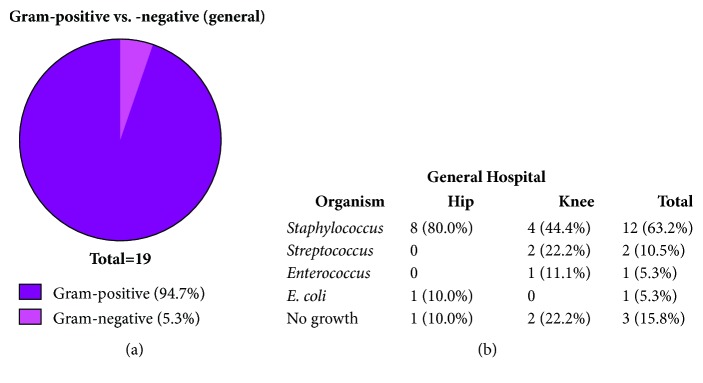
*At general university hospital, a majority of infections are caused by Gram-positive organisms.* (a) The percentages of isolated organisms that were Gram-negative, Gram-positive, or neither or experienced no growth were examined at the general university hospital. (b) The bacterial organisms in hip and knee PJIs at the general university hospital were characterized.

**Figure 2:**
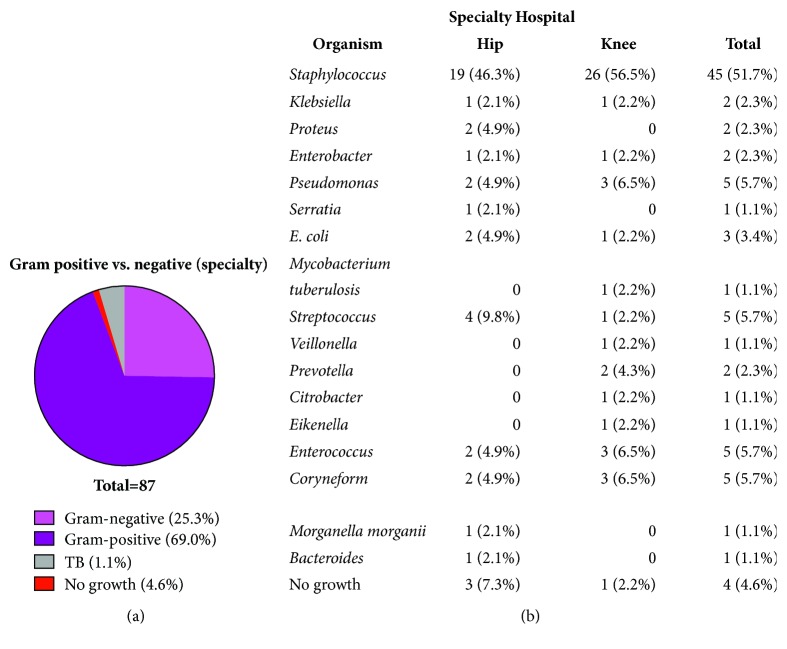
*Increased proportion of Gram-negative PJIs at dedicated orthopaedics hospital.* (a) The percentages of isolated organisms that were Gram-negative, Gram-positive, or neither or experienced no growth were examined at the dedicated orthopaedics specialty hospital. (b) The bacterial organisms in hip and knee PJIs at the dedicated orthopaedics specialty hospital were characterized.

Next, the characteristics of the bacterial organisms cultured from patients with PJIs at the general university were compared to those at the dedicated orthopaedics specialty hospital, as Gram-positive bacteria have been most commonly implicated in PJIs [4–6]. At the general university hospital only 5.3% of cultured organisms were Gram-negative (Figure 1(a)). Interestingly, while 69.0% of cultured organisms from the PJI patients at the dedicated orthopaedics specialty hospital were Gram-positive, 25.3% of cultured organisms were Gram-negative (Figure 2(a)). Importantly, while the majority of cultured organisms from PJIs at both the dedicated orthopaedics specialty hospital and general university hospital were* Staphylococcus *species (Figures 1 and 2) with no statistically significant difference in the diversity of bacterial organisms in either setting (*p*=0.92), and there was a noticeable contrast in the variety of bacterial organisms isolated from the general university hospital compared to the dedicated orthopaedics specialty hospital. In particular, there were 3 bacterial organisms isolated from PJIs at the general university hospital (Figure 1(b)), compared to 17 different bacterial organisms isolated from PJIs at the dedicated orthopaedics specialty hospital (Figure 2(b)). More specifically, from PJIs at the dedicated orthopaedics specialty hospital, there were a variety of Gram-negative species isolated, such as* Klebsiell*a,* Proteus*,* Pseudomonas*,* Serratia*,* E. coli*,* Bacteroides*,* Eikenella*,* Morganella,* and* Veilllonella*, among others (Figure 2(b)). These results suggest that while the overall incidence of PJIs at a dedicated orthopaedics specialty hospital is reduced compared to a general university hospital, there was a nonstatistically significant trend towards an increased percentage of Gram-negative infections (*p*=0.15), with a greater variety of bacterial organisms isolated from PJIs.

**Figure 3:**
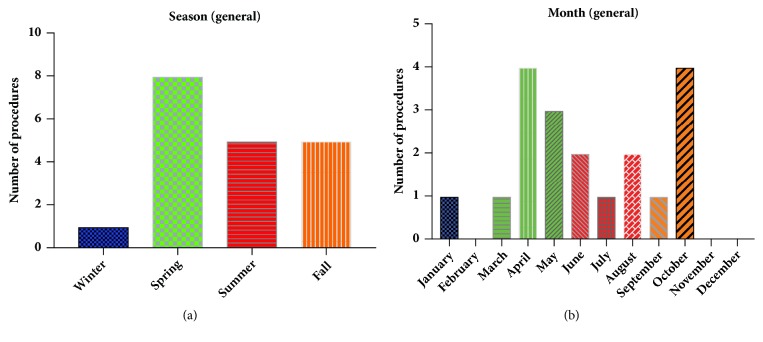
*No trends in seasonality nor procedure month at general university hospital.* (a) The season during which procedures resulting in PJIs at the general university hospital were performed is shown. (b) The particular month during which procedures resulting in PJIs at the general university hospital were performed is shown, with the months color-coded corresponding to the season.

**Figure 4:**
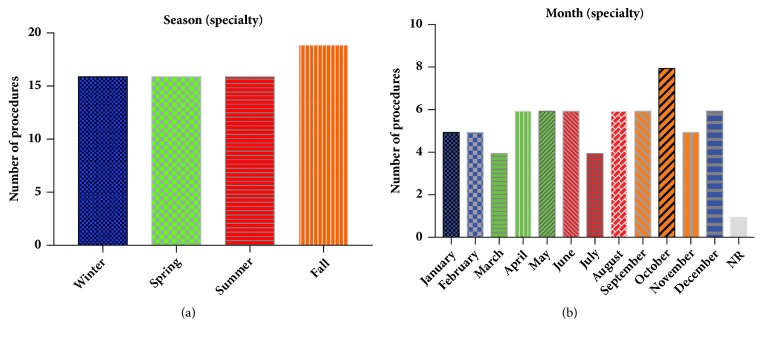
*No trends in seasonality nor procedure month at dedicated orthopaedics hospital*. (a) The season during which procedures resulting in PJIs at the dedicated orthopaedics specialty hospital were performed is shown. (b) The particular month during which procedures resulting in PJIs at the dedicated orthopaedics specialty hospital were performed is shown, with the months color-coded corresponding to the season.

Finally, infection season and month have been correlated to an increased risk of PJIs, with summer months accounting for more PJIs compared to winter months [25, 26]. When examining procedure season and month at the general university hospital, there was a trend towards more infections occurring from procedures performed in the spring compared to other seasons (Figure 3(a)), with the most infections occurring in October and April (Figure 3(b)). However, at dedicated orthopaedics specialty hospital, there was a trend towards an increase in PJIs from procedures that occurred during the fall (Figure 4(a)) and during October (Figure 4(b)). When the procedure month and season between the general university and specialty orthopaedic hospital were directly compared by X^2^ analysis, neither were significantly different (*p*=0.72 and* p*=0.22 for the procedure month and season, resp.).

## 4. Discussion

### 4.1. The Transition from General University to Dedicated Orthopaedics Hospital

Although the transition from general hospitals to specialized hospitals has existed for over 100 years with the 1857 opening of a dedicated cardiac hospital in London [34], over the recent years in the United States, there has been a growing interest in physician-owned hospitals offering specialty care. While only 100 specialty hospitals existed in 1990, that number more than tripled by 2005 [35]. In particular, there has been an increase in specialty cardiac and orthopaedic hospitals. Studies of these specialty hospitals have shown that specialty hospitals generally have better outcomes than general hospitals. A 2005 study showed patients who underwent percutaneous coronary intervention and coronary artery bypass grafting (CABG) at cardiac specialty hospitals were less likely to have associated complications of myocardial infarction and had lower odds ratio for death after CABG and lower readmission rates when compared to general hospitals [36]. Furthermore, Heinemann et al. demonstrated that spinal cord injury (SCI) patients treated at a specialized SCI center had greater daily functional gain compared to a general hospital [37]. Patients at one specialty cardiac specialty hospital had a 12.1% decrease in in-hospital mortality rate compared to patients in a general community and teaching hospitals, indicating that specialized care may provide a wide range of benefits to patients to patients in terms of morbidity and mortality. In orthopaedics specifically, Greenwald et al. found lower overall mortality and readmission rates at the orthopaedic specialty hospitals they examined [38]. However, few studies exist to examine the specific differences among care in orthopaedics specifically at a specialty versus general hospital. Additionally, there is little understanding of how the transition to a dedicated orthopaedics hospital may impact infection rates. Therefore, our study addresses a critical gap in understanding how the infection rates and microbiologic characteristics may differ between a general university hospital and dedicated orthopaedic specialty hospital. In particular, we found an overall lower infection rate at the dedicated orthopaedics specialty hospital with a greater bacterial diversity and higher rate of Gram-negative infections.

While seasonality could certainly impact the increased incidence of Gram-negative PJIs at the dedicated orthopaedics specialty hospital, given that prior studies showed an increased incidence of Gram-negative infections in the summer months [25, 26], we do not interpret our results to indicate that seasonality impacts the incidence of Gram-negative infections observed at the dedicated orthopaedics specialty hospital compared to the general university hospital. While it is interesting to note the seasonal differences in our two cohorts, given the small sample sizes and minor differences, we cannot reasonably conclude that seasonality impacts the variety of Gram-negative infections observed at the dedicated orthopaedic specialty hospital.

### 4.2. Gram-Negative Infections and PJIs

Although a lower overall infection rate at the dedicated orthopaedics specialty hospital was observed compared to the general university hospital, there was a greater variety of Gram-negative bacteria among the PJIs at the specialty hospital. While much remains to be determined regarding Gram-negative PJIs in the orthopaedic literature, as the most common organisms implicated in PJIs are* Staphylococcus* species [39], some studies have detailed approaches for combatting Gram-negative PJIs in particular settings. Zmitowski et al. reported 31/277 (11.2%) of PJIs as colonized with Gram-negative bacteria, with* E. coli* being the most common pathogen; other colonizers included* Proteus*,* Serratia, *and* Klebsiella* [40]—similar pathogens were observed in this series, but at a higher proportion of Gram-negative infections. Furthermore, given that Zmitowski et al. described Gram-negative organisms resistant to third-generation cephalosporins, ciprofloxacin, or gentamicin, when applied to this study, those findings potentially suggest that the Gram-negative bacteria reported in our study may have been resistant to current prophylactic regimens consisting primarily of cephalosporins. Antimicrobial resistance is a particular issue in Gram-negative infections, and, in 2009, Martinez-Pastor et al. reported 47 cases of PJIs due to Gram-negative bacteria, with the most frequent pathogens being members of the* Enterobacteriaceae* family and* Pseudomonas* species, with some resistance occurring to ciprofloxacin in the* Enterobacteriaceae* family [41]. One case report of PJI following total hip arthroplasty found* Clostridium perfingens*, despite receiving intravenous cefuroxime [42], again indicating that prophylactic administration of cephalosporins may not prevent the development of Gram-negative PJIs. In another retrospective observational study, 242 cases of Gram-negative PJIs were reported over a 7-year timespan with* Enterobacteriaceae *(78%) and* Pseuduomonas* species (20%) as the most common isolates, with 19% of isolates being ciprofloxacin-resistant [43]. Together, these previous reports of Gram-negative PJIs, while limited in scope, indicate that even prophylactic administration of cephalosporins may not provide sufficient coverage for all PJI-causing pathogens, particularly in the case of some Gram-negative bacteria. Further studies will be necessary to characterize the prevalence of Gram-negative infections in other hospital settings, and specifically in the rising number of orthopaedic specialty hospitals.

Furthermore, over the time period in which our study was conducted from 2008 to 2016, several new techniques for identifying clinical bacterial isolates have become more widely utilized in clinical laboratories. Standard protocols involve obtaining culture specimes intraoperatively through swabs and tissue samples, followed by sterile transport to a clinical microbiology laboratory for processing and culture. Newer methods include amplifying bacterial genomes of interest through polymerase chain reactions (PCR) or sequencing of bacterial genomes, known as 16S rRNA sequencing for targeting prokaryote rather than eukaryote genome, or alternatively mass spectrometry and 16S rRNA quantitative PCR to estimate bacterial load [44–46]. Although these advances in isolating clinical bacterial species could theoretically contribute to differences of bacterial species identified over the same timeframe if these newer sensitive tests were utilized in the specialty hospital and not the general hospital, given that our study relied on culture results in both settings, differences in these methods are not applicable to the results presented here.

### 4.3. Strengths and Limitations

This study provided a unique opportunity to examine one university system in its transition from orthopaedics practice at a general university hospital to a dedicated orthopaedic specialty hospital. By studying one university system, rather than comparing a community hospital to a separate specialty hospital, we attempted to control for many factors. Although the operative venue changed in 2008, there was no change in the surgeons, residents, patient population served, or discrete changes in surgical technique or irrigation solution, including betadine washes, thus minimizing confounding variables. Despite this strength, given the rare occurrence of infection it is difficult to perform in-depth statistical analysis to elucidate risk factors for infection within this sample. Additionally, as there has been other infection control measures to reduce infection over the past decade it would be speculative to presume that the transition to a dedicated specialty hospital is the sole reason for the reduced infection rate. Additional benefits to reduce infection at a dedicated orthopaedic hospital can include streamlined anesthesia teams and sterile supply processing with dedicated OR staff resulting in shorter OR times, as shown in Table 3, and consistent attention to sterility needed for arthroplasty cases. Additionally, although we are unable to quantify measures of stricter adherence to Surgical Care Improvement Project guidelines or reduction in OR traffic, the consistent attention to sterility is paramount for total joint replacements. Finally, as this study examined the transition from a general hospital to a specialty hospital within an academic setting that has a large catchment area with a diverse patient population, we acknowledge that these results may not be translatable to other practice settings.

### 4.4. Conclusions and Future Directions

In this study, the infection rates and demographic and microbiologic characteristics between patients with PJIs at a general university hospital, were compared to the PJIs after the same university system transitioned to a dedicated orthopaedic specialty hospital. There was a decreased overall infection rate; however, a greater proportion of infections were from Gram-negative organisms with a variety of Gram-negative organisms isolated. These results indicate a potential nuance in dedicated orthopaedic specialty hospital care and need to further study infection prevention and treatment strategies to continue to minimize PJI.

## Figures and Tables

**Table 1:** *Overall incidence rates decreased at dedicated orthopaedics hospital.* Overall incidence rate of PJIs between the general university hospital and dedicated orthopaedics hospital. *p* = .0224 by X^2^ analysis.

	Infected	Overall	Incidence
General university hospital	19	1325	1.43%
Dedicated orthopaedics hospital	68	9055	0.75%

**Table 2:** *2-year incidence rates decreased at dedicated orthopaedics hospital.* Incidence rate of PJIs over a 2-year timeframe between the general university hospital and dedicated orthopaedics hospital. *p* = .0087 by X^2^ analysis.

	Infected	Overall	Incidence
General university hospital	19	1325	1.43%
Dedicated orthopaedics hospital	17	2787	0.61%

**Table 3:** In cases that developed an infection, there was a reduced surgery time for cases performed at the dedicated orthopaedics hospital compared to the general university hospital.

	Surgery time (minutes)
General university hospital	146.08
Dedicated orthopaedics hospital	131.31

**Table 4:** *Demographic variables at general university versus dedicated orthopaedic hospital.* Demographic variables, such as BMI, sex, diabetes, whether a resident/fellow was scrubbed, positive history of steroid use, and smoking status were examined at the general university versus dedicated orthopaedics specialty hospitals. NR = not reported. For comparisons of age and BMI, Mann–Whitney *U* nonparametric analysis utilized. For all other variables, X^2^ analysis utilized.

	**General**	**Specialty**	***p*-value**
Age			
Mean	64.4	61.6	0.13

BMI	30.5	31.5	0.52

Sex			0.43
Male	12 (63.2%)	36 (52.9%)	
Female	7 (36.8%)	32 (47.1%)	

Diabetes	5 (26.3%)	12 (17.6%)	0.40

Resident/fellow scrubbed	11 (57.9%)	43 (63.2%)	0.67

Positive history of steroid use	2 (10.5%)	3 (8.3%)	0.31

Smoker			0.37
Never	11 (57.9%)	44 (64.7%)	
Former	2 (10.5%)	13 (19.1%)	
Current	4 (21.1%)	9 (13.2%)	
NR	2 (10.5%)	2 (2.9%)	

**Table 5:** *Demographic variables at general university versus dedicated orthopaedic hospital for TKA PJIs.* Demographic variables, such as BMI, sex, diabetes, whether a resident/fellow was scrubbed, positive history of steroid use, and smoking status were examined at the general university versus dedicated orthopaedics specialty hospitals. NR = not reported. *p *value was calculated by X^2^ analysis.

	**General**	**Specialty**	***p *value**
Sex			0.90
Male	5 (62.5%)	21 (60%)	
Female	3 (37.5%)	14 (40%)	

Diabetes	3 (37.5%)	5 (14.3%)	0.13

Resident/fellow scrubbed	2 (25.0%)	24 (68.6%)	0.02

Positive history of steroid use	2 (25.0%)	2 (5.7%)	0.09

Smoker			0.04
Never	6 (75.0%)	20 (57.1%)	
Former	0 (0.0%)	9 (25.7%)	
Current	1 (12.5%)	6 (17.1%)	
NR	1 (12.5%)	0 (0.0%)	

**Table 6:** *Demographic variables at general university versus dedicated orthopaedic hospital for THA PJIs.* Demographic variables, such as BMI, sex, diabetes, whether a resident/fellow was scrubbed, positive history of steroid use, and smoking status were examined at the general university versus dedicated orthopaedics specialty hospitals. NR = not reported. *p *value was calculated by X^2^ analysis.

	**General**	**Specialty**	***p *value**
Sex			0.30
Male	7 (63.6%)	15 (45.5%)	
Female	4 (36.4%)	18 (54.5%)	

Diabetes	2 (18.2%)	7 (21.2%)	0.83

Resident/fellow scrubbed	9 (81.8%)	19 (57.6%)	0.15

Positive history of steroid use	0 (0.0%)	1 (3.0%)	0.56

Smoker			0.24
Never	5 (45.4%)	24 (72.7%)	
Former	2 (18.2%)	4 (12.1%)	
Current	3 (27.3%)	3 (9.0%)	
NR	1 (9.1%)	2 (6.0%)	

## Data Availability

The data used to support the findings of this study are included within the article.
